# Insights into Persistence Mechanisms of a Zoonotic Virus in Bat Colonies Using a Multispecies Metapopulation Model

**DOI:** 10.1371/journal.pone.0095610

**Published:** 2014-04-22

**Authors:** Margarita Pons-Salort, Jordi Serra-Cobo, Flora Jay, Marc López-Roig, Rachel Lavenir, Didier Guillemot, Véronique Letort, Hervé Bourhy, Lulla Opatowski

**Affiliations:** 1 Institut Pasteur, Unité de Pharmaco-épidémiologie et Maladies Infectieuses, Paris, France; 2 INSERM, U657, Paris, France; 3 Univ. Versailles−Saint-Quentin-en-Yvelines, EA 4499, Faculté de Médecine Paris Île-de-France Ouest, Garches, France; 4 Centre de Recerca en Infeccions Víriques de les Illes Balears, Palma de Mallorca, Spain; 5 Institut de Recerca de la Biodiversitat, Departament de Biologia Animal, Universitat de Barcelona, Barcelona, Spain; 6 Department of Integrative Biology, University of California, Berkeley, California, United States of America; 7 Institut Pasteur, Unité Dynamique des Lyssavirus et Adaptation à l'Hôte, National Reference Centre for Rabies, WHO Collaborative Centre for Reference and Research on Rabies, Paris, France; 8 AP–HP, Hôpital Raymond-Poincaré, Garches, France; 9 École Centrale Paris, Laboratoire de Mathématiques Appliquées aux Systèmes, Châtenay-Malabry, France; Imperial College London, United Kingdom

## Abstract

Rabies is a worldwide zoonosis resulting from *Lyssavirus* infection. In Europe, *Eptesicus serotinus* is the most frequently reported bat species infected with *Lyssavirus*, and thus considered to be the reservoir of European bat *Lyssavirus* type 1 (EBLV-1). To date, the role of other bat species in EBLV-1 epidemiology and persistence remains unknown. Here, we built an EBLV-1−transmission model based on local observations of a three-cave and four-bat species (*Myotis capaccinii, Myotis myotis, Miniopterus schreibersii, Rhinolophus ferrumequinum*) system in the Balearic Islands, for which a 1995–2011 serological dataset indicated the continuous presence of EBLV-1. *Eptesicus serotinus* was never observed in the system during the 16-year follow-up and therefore was not included in the model. We used the model to explore virus persistence mechanisms and to assess the importance of each bat species in the transmission dynamics. We found that EBLV-1 could not be sustained if transmission between *M. schreibersii* and other bat species was eliminated, suggesting that this species serves as a regional reservoir. Global sensitivity analysis using Sobol's method revealed that following the rate of autumn−winter infectious contacts, *M. schreibersii*'s incubation- and immune-period durations, but not the infectious period length, were the most relevant factors driving virus persistence.

## Introduction

Bats are a group of mammals found worldwide and exhibiting high species diversity (more than 1100 species). They are also the only mammals that can fly and have been found to be a continuing source of emerging viral diseases, including rabies. Rabies is a zoonosis resulting from *Lyssavirus* infection [1]. To date, at least 10 *Lyssavirus* species have been isolated from bats [2]. Interest in identifying the factors enabling virus persistence in bat colonies is growing [3]–[6]. These mechanisms probably depend on bat species' ecology and ethology (habitats, life cycles, colony sizes, species richness) [7] that vary markedly across geographical zones. Identifying key host species for virus persistence and the biological traits that make them important is challenging, but essential, for the design of control strategies, evaluating the risk of human exposure, and predicting the impact of potential ecological disturbances.

**Figure 1:**
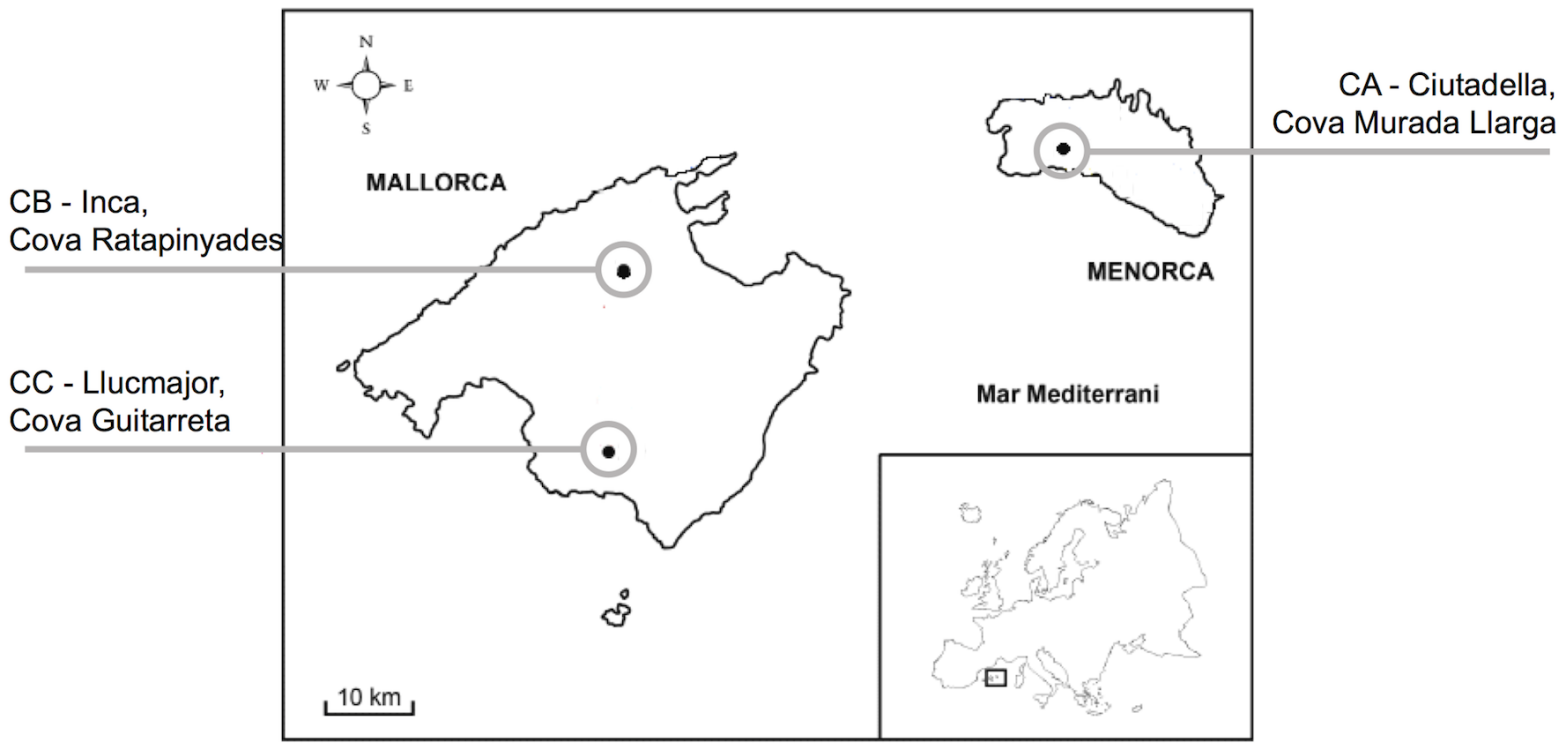
Map of Mallorca and Menorca. Map of Mallorca and Menorca (in the Balearic Islands) and the locations of the caves (CA, CB and CC) considered in the system studied.

European bat *Lyssavirus* type 1 (EBLV-1) is widespread in Europe [8], [9] and can occasionally infect non-flying mammals, including humans [2], [10]. Because most of the bats found infected with EBLV-1 through passive surveillance are *Eptesicus serotinus* [11], the serotine bat is considered the main reservoir for EBLV-1. Serotines are relatively sedentary bats that feed predominantly on large insects in open sheltered urban and parkland areas, mostly in lowlands. They most often roost in buildings in small groups or individually. To date, the role of other bat species in EBLV-1 epidemiology and persistence remains unknown. Here, we focused on a particular system of multispecies bat colonies in the Balearic Islands, Spain (Figure 1), that has been studied for 16 years during which *E. serotinus* was never observed, and we used mathematical modelling to gain insights into the role of other bat species in EBLV-1 persistence.

**Figure 2:**
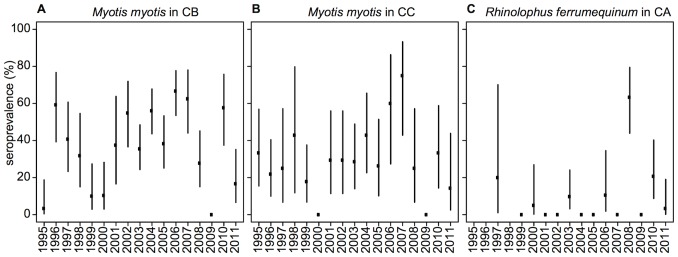
EBLV-1 seroprevalence by subpopulation. Observed EBLV-1 seroprevalences with 95% CI in two *Myotis myotis* colonies (A, B) and one *Rhinolophus ferrumequinum* colony (C). Null rates of seroprevalence at some times might not be indicative of local virus extinctions, but just virus extinctions in that subpopulation or a consequence of under-reporting.

The system studied here comprises four temperate and gregarious bat species (*Myotis capaccinii, Myotis myotis, Miniopterus schreibersii* and *Rhinolophus ferrumequinum*) living in three caves and having a metapopulation structure (spatially discrete subpopulations with seasonal interactions of some individuals). The ecology of these colonies has been studied for >16 years [12], with the first EBLV-1−seropositive bats found in this region in 1995 [13], [14]. Since then, EBLV-1 dynamics in several colonies has been monitored [15]. Long-term serological data on two Mallorcan *Myotis myotis* colonies and one Menorcan *Rhinolophus ferrumequinum* colony revealed high anti-EBLV-1−antibody seroprevalence (Figure 2) [13]. Those findings suggest that EBLV-1 has been circulating in the Balearic Islands at least that long and, thus, is probably enzootic in the region. However, the factors underlying this long-term persistence remain poorly understood. The supposed short duration of the infectious period, together with the expected transmission bottleneck experienced in winter should favour rapid EBLV-1 extinction.

In temperate regions, the four bat species considered have different ecological requirements depending on the season, coinciding with breeding, mating and hibernation [12]. Consequently, summer and winter shelters often differ and bat populations move seasonally between them, thereby connecting seemingly isolated populations. The metapopulation structure, social organization within the refuges (intra- and interspecific interactions), where multiple bat species usually cohabit, and the seasonal behaviour of temperate bats can play important roles in the dynamics of virus persistence. Therefore, integrating a community perspective of several host species and their interactions is crucial to address the problem of virus persistence. To this aim, we developed a metapopulation model describing EBLV-1−transmission dynamics in the system of multispecies bat colonies previously described and based on 16 years of ecological and epidemiological observations (1995–2011). The model was used to analyse the plausibility of several factors that could explain EBLV-1 persistence, including: interisland exchanges of some bat species, seasonality of infectious contacts, and epidemiological characteristic differences among bat species.

## Materials and Methods

### Ethics statement

All animals were handled in strict accordance with good animal practices, as defined by current European legislation. Bat capture and blood-sampling were authorized by the Spanish Regional Committee for Scientific Capture.

### Data collection

Bat ecological and epidemiological data were obtained by capture−recapture of ringed animals. Capture, sampling, colony-size estimation and detection of EBLV-1−neutralizing antibodies in bat sera are described elsewhere [7].

### Bat species and subpopulations

This study's descriptive unit is the *subpopulation*, defined as all the individuals of the same bat species that coexist in a colony. The system studied comprises 10 bat subpopulations of four gregarious species (*M. capaccinii*, *M. myotis, M, schreibersii* and *R. ferrumequinum*) living in three Balearic Island cave-dwelling colonies: one in Cova Murada-Llarga (CA), located in Ciutadella, Menorca, and the two others in Mallorca: Cova Ratapinyades (CB), in Inca, and Cova Sa Guitarreta (CC), in Llucmajor (Figure 1). The minimum distance between the Mallorca and Menorca coasts is 40–50 kilometres.

**Figure 3:**
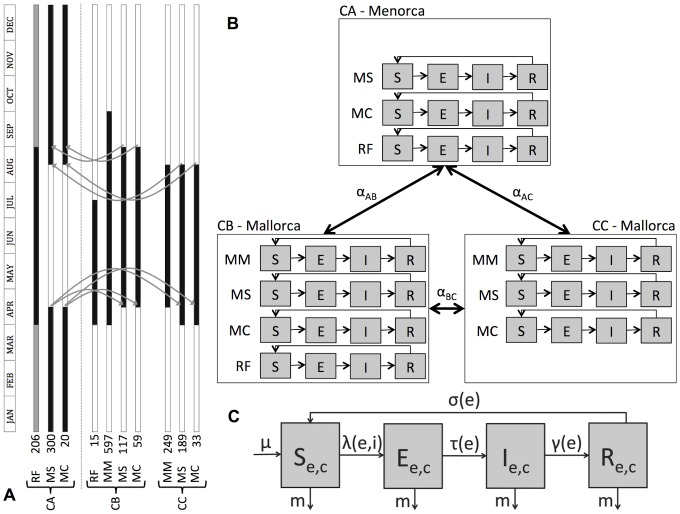
Model description. (A) January to December occupation of the caves by each species (black). The number of individuals of each subpopulation at the beginning of the simulations is indicated on the left. Gray periods represent only one-third of the colony remaining in the cave. Gray arrows indicate the seasonal interisland exchanges of individuals at certain times. Bat species names are abbreviated: MC, *Myotis capaccinii*; MM, *Myotis myotis*; MS, *Miniopterus schreibersii* and RF, *Rhinolophus ferrumequinum*. (B) Schematic representation of the metapopulation model, with three patches corresponding to the caves (CA, CB and CC) considered. Black arrows represent exchanges between caves. Each subpopulation is compartmentalized according to its infectious status: S (Susceptible), E (Exposed), I (Infectious) and R (Recovered). (C) SEIRS-model structure for EBLV-1−infection dynamics in each subpopulation.

These temperate bat subpopulations occupy different caves throughout the year, according to specific ecological requirements. Basically, they roost in a reproduction cave during the spring−summer months, and in a hibernation refuge during autumn−winter. Bats faithfully return each year to their birth and winter refuges. Figure 3a illustrates the pattern of cave occupation by each species. We briefly describe below the annual rhythm and relevant behavioural characteristics of each analysed species obtained by capture−recapture of ringed animals (capture, sampling, methods for colony-size estimation and detection of EBLV-1−neutralizing antibodies in bat sera are described elsewhere [7], [14], [16]). These specificities are also summarized in Table 1 and each bat species' distribution is shown in Figure S1.

**Table 1:** **Bat species characteristics.**

	*Myotis myotis*	*Myotis capaccinii*	*Miniopterus schreibersii*	*Rhinolophus ferrumequinum*
**Common name**	Greater mouse-eared bat	Long-fingered bat	Common bent-wing bat	Greater horseshoe bat
**Family**	*Vespertilionidae*	*Miniopteridae*	*Vespertilionidae*	*Vespertilionidae*
**Suborder ** [18]	Yangochiroptera	Yangochiroptera	Yangochiroptera	Yinpterochiroptera
**Distribution ** [12]	Mallorca	Mallorca & Menorca	Mallorca & Menorca	Mainly Menorca
**Refuge ** [12]	CB, CC	CA, CB, CC	CA, CB, CC	CA, CB
**Movements ** [17]	Sedentary	Migratory	Migratory	Sedentary
**Hibernation**	Isolated-small groups	Winter refuges with maintained continuous activity	Winter refuges with maintained continuous activity	1/3 of the CA colony remains in the cave; the rest are isolated

*M. myotis* is widespread throughout Mallorca, but has never been observed in Menorca. Its behaviour is mainly sedentary and with short on-island displacements. From spring to late summer, *M. myotis* forms breeding colonies in caves or rock crevices. From late summer to spring, this species forms small isolated groups.

*M. schreibersii*, a regional migratory bat species with relatively fast flight capable of making long journeys [16], [17], is found on Menorca and Mallorca. In winter, it forms big colonies and remains relatively active during this period. On Menorca, in late-winter, *M. schreibersii* moves to breeding colonies in the south coast. No winter colonies have ever been found on Mallorca. It is thought that a few individuals forming breeding colonies in Inca (CB) and Llucmajor (CC) migrate to winter on Menorca, where they join the *M. schreibersii* previously described [16]. The migrating individuals would return to Mallorca in spring.

*M. capaccinii* is a regional migrant [16], [17], whose distribution is strongly linked to *M. schreibersii*. In the refuges, it usually lodges among *M. myotis* and *M. schreibersii*.

*R. ferrumequinum* lives predominantly on Menorca, although some small colonies live on Mallorca. It is a sedentary species. In winter, it normally forms small groups, although CA (Menorca) shelters a winter colony of about 80 individuals. *R. ferrumequinum* belongs to the family *Rhinolophidae* of the suborder Yinpterochiroptera, while the three other bat species described above are of suborder Yangochiroptera [18].

Refuges CB and CC shelter breeding colonies of *M. myotis*, *M. schreibersii* and *M. capaccinii* (and a small number of *R. ferrumequinum* in CB) from spring to autumn. CA harbours a large *R. ferrumequinum* colony year-round, serves as intermediate refuge for *M. schreibersii* and *M. capaccinii* during their seasonal migrations between hibernation and breeding shelters, and the latter two continuously move back and forth between CA and their Menorcan winter colony.

All the subpopulations considered here were sampled and seropositive individuals identified for each. Long-term serological data for *M. myotis* and *R. ferrumequinum* are shown in Figure 2. For *M. schreibersii* and *M. capaccinii*, serological data were collected only the last few years and are not shown.

### Ecological factors

Two ecological factors that could play a role in EBLV-1 persistence are included in the model. The first factor was the seasonal interisland exchanges of migratory *M. schreibersii* and *M. capaccinii* at certain times: from Mallorca to Menorca in late summer, and vice versa at early spring (Figure 3a). This phenomenon was reported by Amengual *et al*. who observed some individuals ringed on one island later being recaptured on the other [16]. Additionally, phylogenetic studies show that viral lineages circulating on Mallorca and Menorca are indistinguishable, possibly indicating an interisland exchange [7], [19]. The second factor was seasonality of contact rates due to the winter period. In the Balearic Islands, autumn and winter environmental conditions are not extremely harsh, and, consequently, bats do not truly hibernate, remaining active during those months. Nevertheless, intra- and interspecific contact rates probably change from high (breeding season) to low (autumn−winter). Specifically, *M. myotis* forms isolated small groups during winter, thereby eliminating contact with other bat species.

### Model

Using the bat ecology described above, we developed a metapopulation model describing the EBLV-1−circulation dynamics in those colonies. The model was used to examine determinants of EBLV-1 persistence through three different approaches. First, we explored the influence of ecological factors: seasonal interisland exchanges and seasonality of contacts, on EBLV-1 persistence. Second, we tested the role of each bat species as a potential key host for EBLV-1 persistence. Lastly, we applied a global sensitivity-analysis method to identify the most influential factors (including ecological and epidemiological) on EBLV-1 persistence.

### Metapopulation structure

The metapopulation model includes three patches representing the three caves (Figure 3b). The patches are connected through seasonal *M. schreibersii* and *M. capaccinii* interisland exchanges at two times: late summer (end of maternity colonies) from Mallorca to Menorca, and just before early spring for breeding from Menorca to Mallorca (Figure 3a). The number of individuals of each subpopulation is fixed using the data reported in [19] (Figure 3a).

### Demographic and infection dynamics within a population

The EBLV-1−infection dynamics within each subpopulation is described by a classical SEIRS model (Figure 3c). Newborns enter the model as susceptible (*S*) individuals, at rate µ, equal to the death rate *m* to maintain a constant population size. Birth and mortality rates are assumed to be constant over the year.

Susceptible individuals when they are in contact with infectious individuals can become infected but not yet infectious (*E*), representing the incubation period. After an average time of 1/*τ* days, infected individuals become infectious (*I*) and mount an immune response at rate *γ* and move to recovered (*R*) status. Because immunity is not lifelong, individuals again become susceptible, returning to the S compartment after an average of 1/*σ* days. The average incubation (1/*τ*), infectious (1/*γ*) and immune periods (1/*σ*) are parameters dependent on the host species. Moreover, no increased mortality due to infection was included. All model parameters are summarized in Table 2.

**Table 2:** **Parameter values.**

Ecological factors			
Interisland exchange rate	− (0.0–0.1)		
Contact reduction, *r*	− (0.0–1.0)		
**Demographic factors**			
Birth rate, µ	0.1 years^−1^		
Death rate, *m*	0.1 years^−1^		
**Epidemiological factors**	**MM**	**RF**	**MS & MC**
Intraspecies *R*_0_	− (1–5)	− (1–5)	− (1–5)
Incubation-period duration*, τ^−1^	24 (7–58) days	24 (7–58) days	24 (7–58) days
Infectious-period duration**, γ^−1^	7 (4–10) days	10 (7–100) days	7 (4–10) days
Immune-period duration***, σ^−1^	6 (3–60) months	3 (1–12) months	6 (3–60) months

Interval boundaries of the uniform distributions used to sample the 14 uncertain input factors included in the sensitivity analyses (i.e., ecological and epidemiological factors) are given between parentheses: default value (min - max).

*Incubation period. The range was set using data for EBLV-1 reported in [2]. The default value was set to the mean reported in [26] for RABV infection in *Eptesicus fuscus*.

**Infectious period. Observed *Lyssavirus* infectious periods in mammals have not exceeded 1 month [23] and thus it is probably measured in days. Amengual et al., Plos One (2007) estimated the infectious period of *M. myotis* at 5.1 days [13]. A later reevaluation with longer time-series data gave an estimate of approximately 7 days. The uncertainty intervals were set around that value.

***Immune period. Individual data from *M. myotis* recaptured bats in the Balearic Islands suggest that the presence of antibodies might be measured in years [19].

Because no data exist for *M. schreibersii* and *M. capaccinii*, we used the same values that we used for *M. myotis*. Furthermore, for the infectious and the immune periods, the differences between *R. ferrumequinum* and the other three species were set according to population-level observations. In the Balearic Islands it has been observed that *R. ferrumequinum* populations have lower levels of antibodies, but higher levels of viral RNA positive blood clots, than *M. myotis* [19].

### Transmission periods

EBLV-1 is probably transmitted through bites from an infected bat. We defined high- and low-transmission periods depending on the strength of the contact rates. Spring−summer months, in maternity colonies, are assumed to be the high-transmission period, because bats are in close contact inside the caves and are very active. Early-autumn to late-winter is characterized by low or null EBLV-1 transmission, because of decreased activity and some subpopulations isolated in small groups. Therefore, we assumed that a given subpopulation was in the high-transmission period only in CA, CB and CC during the breeding season; otherwise, it was in the low-transmission period.

### Force of infection and contact matrix

Infectious events are assumed to be frequency-dependent [6], [20]. The force of infection at time *t* for a given species *e* that roosts in a cave *c* is the per capita rate of infection acquisition and comprises transmission within and between bat species: 
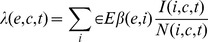
where *β*(*e,i*) is the rate of infectious contacts that depends on the degree of mixing between the infectious species *i*, and the susceptible species *e*, and, consequently, on the transmission periods through different contact matrices (see below). The variables *I*(*i,c,t*) and *N*(*i,c,t*) correspond, respectively, to the number of infectious individuals and the total number of individuals of the species *i* that roost in the cave *c* at time *t*. Note that two subpopulations can be in contact only if they roost in the same cave. Moreover, at a given time, two subpopulations that roost in the same cave (CA, CB or CC) can be in contact only if at that time both occupy the cave or both do not. We defined two contact matrices, *C_h_* and *C_l_*, that summarize the strength of intra- and interspecific contacts during the high- and low-transmission periods, respectively. Such matrices are symmetric and depend only on the species (from left to right and from top to bottom, the coefficients correspond to: *M. myotis, R. ferrumequinum, M. schreibersii* and *M. capaccinii*). Their coefficients were set from qualitative field observations that allowed classification of the degree of interspecies mixing relatively to the intraspecies mixing, into three classes: none (0.0), intermediate (0.5) or close contacts (1.0)



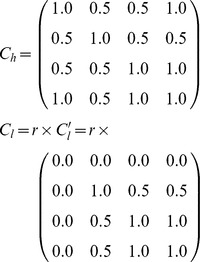
where *r* values fall within [0,1] and represent the reduction of contacts during the low-transmission period. Note that the *C_l_*' matrix simply reflects the absence of *M. myotis* contacts with other species during the low-transmission period, but does not differ from *C_h_* for the other species.

The basic reproduction number is a classical metric in epidemiology that measures the epidemic potential of a pathogen. As the infection and incubation durations are much shorter than life expectancy, here we define the *i*-intraspecies basic reproduction number *R*_0_(*i*) as *β*(*i,i*)/γ(*i*). With this formulation, the rates of intraspecies infectious contacts during high- and low-transmission periods are given by 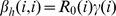 and 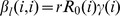 respectively. The rates of interspecies infectious contacts during the high- and low-transmission periods are then deduced: 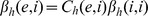 and 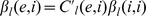 respectively.

### Quantifying EBLV-1 persistence and fade-out

**Figure 4:**
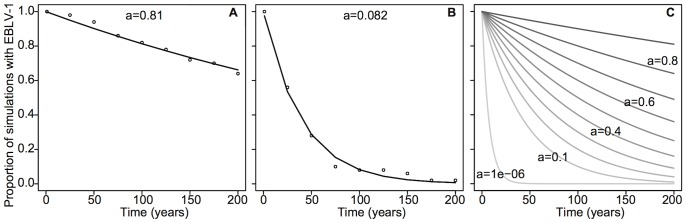
Virus persistence quantification. Fluctuations due to stochasticity cause virus extinction over long times. In order to study virus persistence, we defined the persistence index, *a*. For a given parameter set, we run the model 100 times and estimate *a* by fitting the function *f*(*t*) = *a* ^(0.01*t*)^ to the decreasing proportion of simulations over time with virus persistence. (A, B) The decreasing proportion of simulations with EBLV-1 over time (circles) for two different parameter sets, and the corresponding fit of the function *f*(*t*) = *a* ^(0.01)^ (black line). The persistence index *a* quantifies the probability of EBLV-1 persistence over time. A value of *a* = 0.8 indicates that, after 100 years, EBLV-1 probability of persisting is 0.8. The parameter values used to generate the decreasing curves of simulations with virus persistence (corresponding to those in Mallorca) are the default values reported in Table 2, and for (A), *R*_0_ = 3, *r* = 0.6 and interisland exchange  = 15 individuals, and for (B), *R*_0_ = 3, *r* = 0.5 and interisland exchange  = 3 individuals. (C) *f*(*t*) over 200 years for ten reference values of the persistence index.

Because the bat subpopulations considered are small (orders vary from 10 to 10^3^), we used a stochastic version of the model implemented using the Gillespie algorithm (see Table 3 for a detailed description of the stochastic model). Fluctuations due to stochasticity cause virus extinction over long times. Here, EBLV-1 can be enzootic in the system, and, simultaneously, the number of infected individuals in some subpopulations can also undergo important fluctuations (outbreaks) due to host-ecology seasonality. Therefore EBLV-1 fade-out can be a combination of enzootic and epizootic fade-outs [20]. To study EBLV-1 persistence and fade-out, we introduced a quantity that we call the *persistence index* hereafter. For a given parameter set, we ran the model 100 times over 200 years, thereby generating 100 different trajectories, and recorded the proportion of simulations with persistent virus circulation over time. Intrinsically, those proportions decrease exponentially with a limit of zero as time increases. The persistence index, *a*, is estimated by fitting *f*(*t*)  = *a* ^(0.01*t*)^ to the decreasing curve of the proportion of simulations with virus persistence over time, where *a* falls within ]0,1[ and *t* is the time in years. Thus *f*(*t*) estimates the probability that EBLV-1 will persist at time *t*. In other words, *a* ^0.01^ estimates the probability of virus persistence per year. For example, *a* = 0.8 indicates that, within 100 years, the probability of the virus persisting is 0.8. See Figure 4 for some illustrative examples. The initial prevalence of EBLV-1 was set high enough (2% for *M. myotis*, *M. schreibersii* and *M. capaccinii*, and 10% for *R. ferrumequinum* [19]) to ensure the establishment of the infection in the population. Simulations showed that quasi-stationarity was reached with less than 5 years and that this short burning period did not affect the computation of the persistence index.

**Table 3:** **Model description.**

Events	Transitions	Rates
Birth	*S*(*e*,*c*) → *S*(*e*,*c*)+1	µ*N(*e*,*c*)
Death	*S*(*e*,*c*) → *S*(*e*,*c*)−1	m*S(*e*,*c*)
	*E*(*e*,*c*) → *E*(*e*,*c*)−1	m*E(*e*,*c*)
	*I*(*e*,*c*) → *I*(*e*,*c*)−1	m*I(*e*,*c*)
	*R*(*e*,*c*) → *R*(*e*,*c*)−1	m*R(*e*,*c*)
Infection acquisition	*E*(*e*,*c*) → *E*(*e*,*c*)+1	λ(*e*,*c*,*t*)*S(*e*,*c*)
	*S*(*e*,*c*) → *S*(*e*,*c*)−1	
Incubation to infectious	*I*(*e*,*c*) → *I*(*e*,*c*)+1	τ(*e*)*E(*e*,*c*)
	*E*(*e*,*c*) → *E*(*e*,*c*)−1	
Infection clearance	*R*(*e*,*c*) → *R*(*e*,*c*)+1	γ(*e*)*I(*e*,*c*)
	*I*(*e*,*c*) → *I*(*e*,*c*)−1	
Immunity loss	*S*(*e*,*c*) → *S*(*e*,*c*)+1	σ(*e*)*R(*e*,*c*)
	*R*(*e*,*c*) → *R*(*e*,*c*)−1	
Migration from cave *c1* to cave *c2* #	*S*(*e*,*c1*) → *S*(*e*,*c1*)−1	α(*e*,*c1*,*c2*)*S(*e*,*c1*)
	*S*(*e*,*c2*) → *S*(*e*,*c2*)+1	
	*E*(*e*,*c1*) → *E*(*e*,*c1*)−1	α(*e*,*c1*,*c2*)*E(*e*,*c1*)
	*E*(*e*,*c2*) → *E*(*e*,*c2*)+1	
	*I*(*e*,*c1*) → *I*(*e*,*c1*)−1	α(*e*,*c1*,*c2*)*I(*e*,*c1*)
	*I*(*e*,*c2*) → *I*(*e*,*c2*)+1	
	*R*(*e*,*c1*) → *R*(*e*,*c1*)−1	α(*e*,*c1*,*c2*)*R(*e*,*c1*)
	*R*(*e*,*c2*) → *R*(*e*,*c2*)+1	

List of stochastic events, their transitions and rates for a given species *e* in a given cave *c*. The total population size is *N*(*e*,*c*) = *S*(*e*,*c*)+*E*(*e*,*c*)+*I*(*e*,*c*)+*R*(*e*,*c*), where *S*(*e*,*c*) is the number of susceptible individuals, *E*(*e*,*c*) is the number of infected but non-infectious individuals, *I*(*e*,*c*) is the number of infectious individuals, and *R*(*e*,*c*) is the number of recovered individuals. The force of infection at time *t* for the species *e* in the cave *c* is given by λ(*e*,*c*,*t*), and α(*e*,*c1*,*c2*) is the rate of exchange of species *e* from the cave *c1* to the cave *c2*. The rest of the parameters are defined in Table 2.

# This event is only defined for migratory species (*Miniopterus schreibersii* and *Myotis capaccinii*) during the migratory periods: MS_CA→CB_ (16–30 April), MS_CA→CC_ (16–30 April), MS_CB→CA_ (01–15 September), MS_CC→CA_ (16–30 August), MC_CA→CB_ (16–30 April), MC_CA→CC_ (16–30 April), MC_CB→CA_ (01–15 September), MC_CC→CA_ (16–30 August). For a given simulation, the rates of exchange between caves α(*e*,*c1*,*c2*) and α(*e*,*c2*,*c1*) are such that the same number of individuals migrating from *c1* to *c2* will later move from *c2* to *c1* in order to guarantee that population sizes remain constant over time.

### Sensitivity analysis

To extend the analysis of EBLV-1 persistence to ecological and epidemiological factors, we conducted global sensitivity analyses using Sobol's method [21], [22] with Latin Hypercube Sampling, aiming to establish the relative influence of the model's input factors in the persistence index. The main idea of Sobol's method is to decompose the output variance into the contributions associated with each input factor. Here, we estimated the first- and the total-order sensitivity indexes for each input factor. For a given input factor, the first-order sensitivity index estimates the output sensitivity with respect to this factor individually, whereas the total-order sensitivity index accounts for all the contributions to the output variation due to that factor (i.e. the first-order effect plus all its interactions with other factors). See the Supporting Information S2 for a detailed description of the Sobol method.

The role of 14 uncertain input factors was analysed, assuming uniform distributions for each of them as detailed in Table 2. The parameters included in the analysis were those with the strongest uncertainty, corresponding to those that cannot be directly measured by the ecologists in the field. We included the epidemiological parameters (intraspecies *R*_0_, and the incubation-, infectious- and immune-period durations) for each species (assuming that they are identical for *M. schreibersii* and *M. capaccinii*), seasonal interisland exchanges and the reduction of contacts during the low-transmission period, *r*.

### Model implementation

The stochastic model is described in Table 3, where all the stochastic events, their transitions and rates are listed. The model was simulated using the Gillespie algorithm. The model and the sensitivity analysis were implemented in C. Because of the high number of simulations required to compute Sobol's indexes, sensitivity analyses were parallelized and run on the cluster of Institut Pasteur.

## Results

### Model outputs

**Figure 5:**
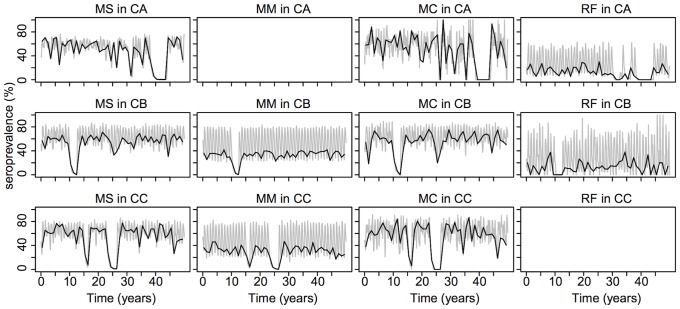
One model simulation run. Gray lines correspond to anti-EBLV-1 antibody seroprevalence in each subpopulation over 50 years. Black lines correspond to seroprevalence measured at the end of May. The parameter values used are: *R*_0_ = 3, *r* = 0.6, interisland exchange  = 3 individuals, immune-period duration  = 6 months, and the default values reported in Table 2 for the incubation- and immune-period durations. Abbreviations are as given in the legend to Figure 3.

The model is able to generate local dynamics of persistence qualitatively similar to reported data (Figure 2). This is illustrated in Figure 5, which presents one random output of the model. Stochastic effects can be observed, with EBLV-1 fade-out at certain times for some subpopulations, followed by virus reintroductions.

EBLV-1 persistence is quantified with the persistence index that allows us to estimate the probability of virus persistence per year. As the examples in Figure 4 demonstrate, the assumption that this probability is constant over time is consistent with simulated data.

### Role of ecological factors in EBLV-1 persistence

We started the analysis of virus persistence on each island by exploring the influence of two ecological factors: seasonal interisland exchanges and reduction of contacts during the low-transmission period (*r* values close to 1 indicate high-contact rates, and those close to 0 indicate low-contact rates). We ran the model varying the strengths of those two factors, while keeping all other parameter values identical across simulations (see default values in Table 2), and three different intraspecies *R*_0_ values: 2, 4 and 6 that, for simplicity, were considered the same for all the bat species.

**Figure 6:**
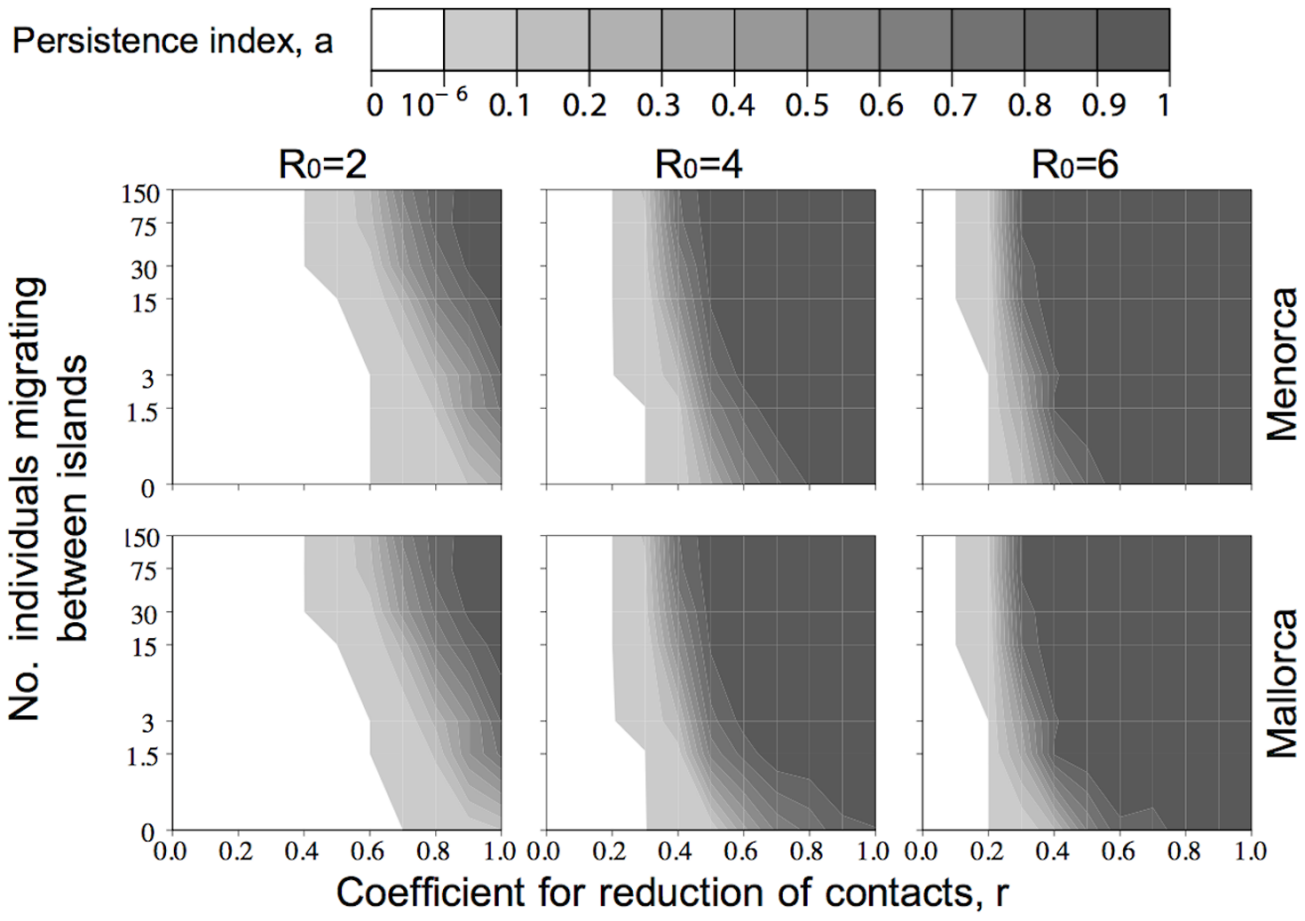
Ecological factors effect in EBLV-1 persistence on both islands. The persistence index is represented as a function of the seasonal interisland exchanges (*Y*-axes) and reduction of contacts during low-transmission periods (*X*-axes), for three different intraspecies *R*_0_ values (2, 4 and 6). Persistence index shades of gray from white to black correspond, respectively, to low and high probabilities of virus persistence.

Figure 6 shows how the persistence index varied on Mallorca and Menorca as a function of the strengths of the two ecological factors. The first thing to note is that, by default (when no contacts are assumed during the low-transmission period, and no interisland exchanges, corresponding to coordinates (0,0) in the six graphs), even a high *R*_0_ could not explain EBLV-1 persistence. As expected, persistence increases with increasing *R*_0_. For the lowest *R*_0_ value, 2, virus persistence seems improbable and would require either a high-contact rate during the low-transmission period (>0.7) or an improbable high number of individuals seasonally moving between islands (>15). For the highest *R*_0_ value, 6, the zone corresponding to a null persistence index changed abruptly to a maximum persistence index, meaning that either EBLV-1 strongly persists or not, with hardly any virus persistence differences between islands. Moreover, the nearly vertical profiles indicate that EBLV-1 persistence is influenced more by the rate of contacts during the low-transmission period than by interisland exchanges.

### Role of the different bat species in EBLV-1 persistence

**Figure 7:**
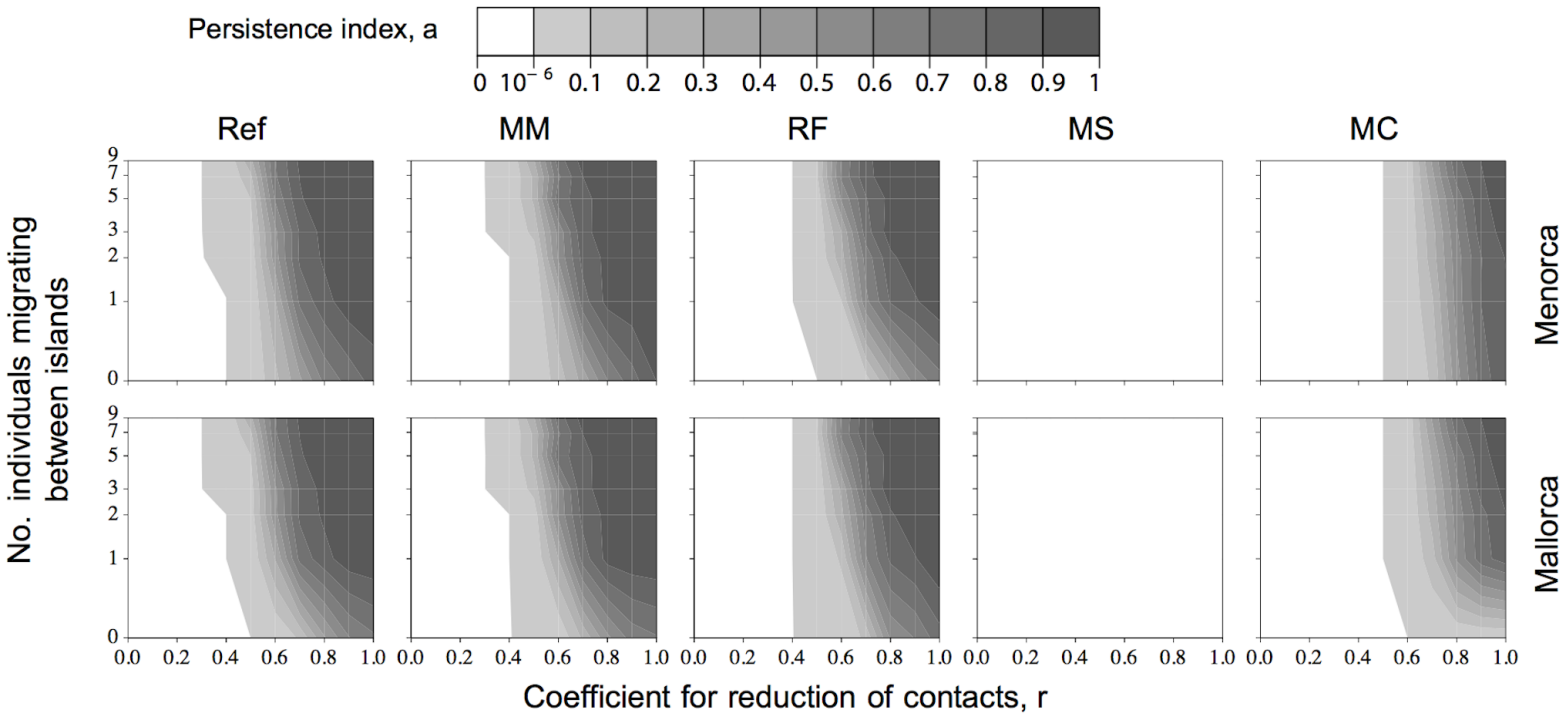
Individual bat species effect in EBLV-1 persistence. The persistence index is represented as in Figure 6. The first column corresponds to the reference scenario (Ref), in which none of the species is removed from the transmission process. The remaining columns correspond to the tested scenarios in which one after the other the indicated species is excluded from the transmission process. Abbreviations are as given in the legend to Figure 3.

To investigate whether each bat species is essential for EBLV-1 persistence, each was removed successively from the transmission process (i.e., individuals cannot acquire nor transmit infections) and compared to the reference scenario (i.e., all species participate on the transmission process), with intraspecies *R*_0_ set at 3 (Figure 7) for all the bat species.

Removing infectious *M. myotis* contacts made no difference on EBLV-1 persistence. Although this observation is self-evident for Menorca, which harbours no *M. myotis*, it was not expected for Mallorca, where *M. myotis* is the most abundant species. It suggests that *M. myotis* does not play a fundamental role in EBLV-1 persistence. Similarly, removing infectious *R. ferrumequinum* or *M. capaccinii* contacts had no consequences on EBLV-1 persistence on either island, whereas removal of infectious *M. schreibersii* contacts made EBLV-1 disappear completely from the system, and even a high *M. capaccinii* contact rate during the low-transmission period and interisland *M. capaccinii* exchanges (corresponding to the top right corner of each graph) could not maintain the virus, probably because *M. capaccinii* subpopulations are small (<60 individuals in CB and CC). These simulation outcomes indicate that *M. schreibersii* is the only essential species for EBLV-1 persistence in the system.

To obtain more insights into how *M. schreibersii* contributes to EBLV-1 persistence, we examined the average enzootic period durations (this term refers to the periods between the introduction of the virus in a population and its fade-out) for each subpopulation in the system, setting *R*_0_ at 3 and *r* at 0.6, with different numbers of individuals transiting between islands. *M. schreibersii* subpopulations had the longest enzootic period, with an average of 25 years in colony CA, and 9 years in colonies CB and CC. In those subpopulations, the average durations of EBLV-1-free periods diminished with increasing numbers of individuals moving between islands, but always lasted a few years (mean <3), which is in accordance with *Myotis myotis* seroprevalence data (Figure 2). These results underscore the major role of *M. schreibersii* in the persistence of EBLV-1 in the system, and suggest that this species serves as a regional reservoir. But so far, we have still not identified the specific biological traits of this species that drive its importance on EBLV-1 persistence.

### Global sensitivity analysis

Uncertainty input-parameter ranges are required to conduct global sensitivity analyses (Table 2). Note that the *R. ferrumequinum* infectious-period interval is particularly long. Indeed, according to field observations, a high proportion of *R. ferrumequinum* individuals' blood clots were viral RNA positive [7]. This peculiarity might be explained by an infectious period for *R. ferrumequinum* longer than that of the other three bat species. Genetic studies showed that the *Rhinolophidae* diverged earlier than *Vespertilionidae* and *Miniopteridae* [18], and, consequently, host−pathogen-coevolution processes could have differed. To take this hypothesis into account and test its impact on EBLV-1 persistence, the *R. ferrumequinum* infectious period duration was allowed to vary from 10 to 100 days.

**Figure 8:**
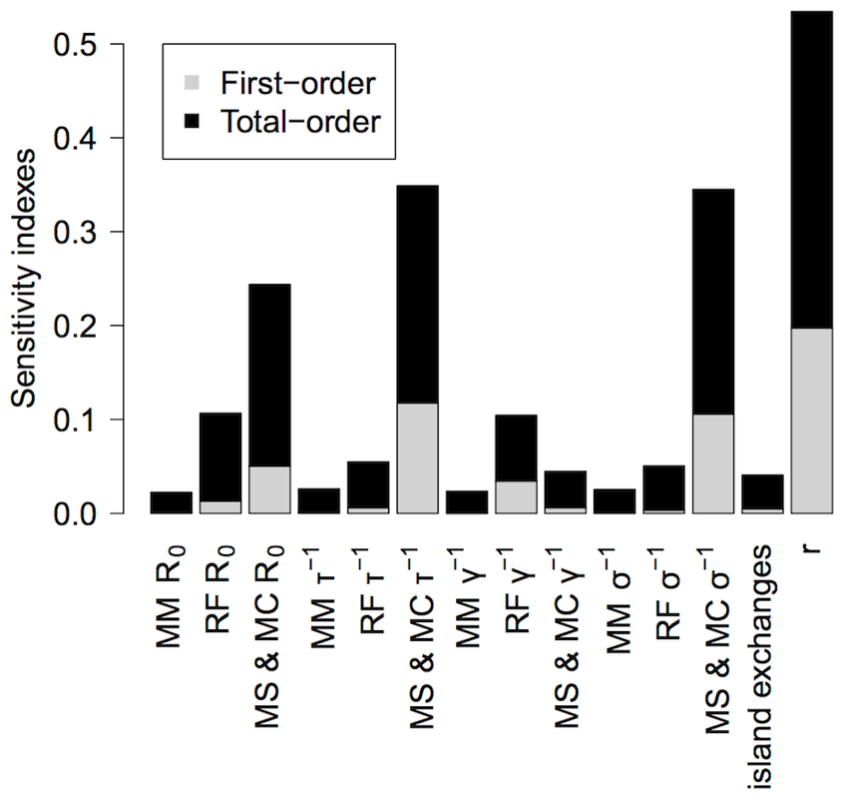
Estimated sensitivities of EBLV-1 persistence-index to the parameters in the model. The persistence index is highly sensitive to the reduction of contacts in the low-transmission period, and three *M. schreibersii* and *M. capaccinii* related parameters (*R*_0_, and incubation- and immune-period durations). Virus persistence is also slightly sensitive to the *R. ferrumequinum R*_0_ and the infectious period duration. The remaining parameters have total-order Sobol' indexes <0.1. Abbreviations are as given in the legend to Figure 3.

The total-order index measures the output sensitivity with respect to an input factor inclusive of its interactions with other parameters. Sensitivity-analysis results for the outcome persistence index calculated over the entire system (Figure 8) showed that the reduction of contacts during the low-transmission period (total-order index  = 0.53) is the parameter that most influences EBLV-1 persistence, in agreement with our previous results (Figure 6). In support of results showing *M. schreibersii*'s crucial role in EBLV-1 persistence (Figure 7), virus persistence was highly sensitive to three *M. schreibersii*-related parameters: incubation- and immune-period durations, and intraspecies *R*_0_, with respective total-order indexes of 0.35, 0.34 and 0.24. Intriguingly, the infectious period seemed not as important as the incubation-period duration. Moreover, the *R. ferrumequinum* infectious period duration, and its *R*_0_ were ranked 5^th^ and 6^th^, suggesting their minor impacts on EBLV-1 persistence. The Sobol' total order indexes for the remaining parameters were <0.1, indicating that they account for <10% of the total persistence-index variance.

It is interesting to note that for all the input factors, the total-order sensitivity index is much higher than the first-order, which indicates the presence of important interactions between factors and thus a highly non-linear model behaviour.

Although sensitivity analysis results showed that a long *R. ferrumequinum* infectious period would not be among the most important factors influencing EBLV-1 persistence, in order to explore how it could specifically impact our initial analysis of the roles of ecological factors and each bat species on EBLV-1 persistence on each island, we ran the same analysis now assuming a hypothetical 50- or 100-day long *R. ferrumequinum* infectious period (Supporting Information S1).

## Discussion

The serotine bat (*E. serotinus*) is by far the most frequently reported bat species infected with *Lyssavirus* in Europe [11], and consequently, it is considered the reservoir of EBLV-1. The role of other bat species on EBLV-1 epidemiology and persistence remains poorly understood. We focused here on a system of three colonies and four bat species (*M. capaccinii*, *M. myotis*, *M. schreibersii*, *R. ferrumequinum*), with refuges on Menorca and Mallorca, for which 1995–2011 serology data indicate the continuous presence of EBLV-1. Serotines have never been observed in the system during the 16-year follow-up. As transmission between species is unlikely to occur outside the roosts, we assumed that transmission cycles in our system were independent from those of *E. serotinus*. We developed a metapopulation model describing EBLV-1 transmission in the system and used it to explore virus persistence mechanisms and to assess the importance of each bat species in the transmission dynamics. Our findings indicate that *M. schreibersii* is the only essential species for EBLV-1 persistence in the system, and suggest it serves as a regional EBLV-1 reservoir. If *R. ferrumequinum* has a longer-than-expected infectious period, which was hypothesized based on field observations but seems unlikely (observed infectious periods in mammals until now have not exceeded 1 month [23]), this species might also play a role in EBLV-1 persistence in the system studied. Further investigations are needed to explore this hypothesis.

Sensitivity-analysis results confirmed the large contribution of *M. schreibersii* to EBLV-1 persistence, inferring that the incubation- and immune-period durations but not the infectious period length, were the most relevant factors after the rate of autumn−winter infectious contacts. Unsurprisingly, the immune-period duration appears relevant, because it controls the replenishment of the susceptible pool (together with the population renewal, i.e. births and deaths). Notably, we found that the incubation-period length (defined here as the time between virus exposure and infectiousness) was one of the most important factors for EBLV-1 persistence. This result must be emphasized, as it improves our understanding of *Lyssavirus* persistence in small metapopulation-structured populations. A relatively long incubation period slows the transmission process, thereby avoiding short-term and large outbreaks, and favouring small-scale infections. Moreover, it increases the chances of an individual exposed to the virus in one place moving to a neighbouring location and introducing the virus into a naïve colony.

There are a number of factors that, surprisingly, were not found to have an essential role in EBLV-1 persistence. First, although *M. Myotis* was the most abundant species (over twice the size of the other species in Mallorca), removing *M. Myotis* infectious contacts did not affect EBLV-1 persistence. Secondly, while we expected that EBLV-1 persistence was explained by between-islands exchanges together with the presence of *R. ferrumequinum* in colony CA year-round (even for a short infectious period), we showed that removing *R. ferrumequinum* infectious contacts did not make any change in EBLV-1 persistence, and between-islands exchanges was not retained as an important factor by the sensitivity analysis. Additionally, removing infectious contacts of *M. capaccinii*, which was by far the least abundant species in the system, had a slight effect on virus persistence, probably because this species roosts within *M. Myotis* and *M. schreibersii* in the caves.

Our analyses revealed that *M. schreibersii* is a key host for EBLV-1 persistence in our setting and suggested that it acts as a regional reservoir. The role of *M. schreibersii* in the transmission of EBLV-1 in Europe remains unknown. In Mediterranean regions, where it is highly abundant and can fly long distances, *M. schreibersii* could also have a critical role in virus persistence, and also in long-distance transmission. These speculations warrant further investigation.

Recently, some authors focused on understanding *Lyssavirus* persistence within other bat species in other settings. George *et al.* (2011) showed that spring birthing, along with hibernation effects (maintaining the system in stasis during the winter) were responsible for bat rabies virus persistence in *Eptesicus fuscus* in Colorado [3]. Hibernation is not a valid mechanism for EBLV-1 persistence in our system, because harsh environmental conditions are unusual in the Balearic Islands, meaning that the four bat species considered do not truly hibernate and maintain some activity throughout the winter months. In contrast, we found that some infectious contacts in winter are needed to observe virus persistence. Hayman *et al*. (2012) hypothesized that the seasonal migratory behaviour of *Eidolon helvum* could be the key factor for Lagos bat virus persistence in two big colonies in Ghana, where hibernation is also absent [4]. In our system, two of the four bat species could be exposed to seasonal interisland exchanges, but that factor was not retained as being relevant for EBLV-1 persistence. However, the impact of bat movements on virus persistence has certainly been underestimated, because only interisland exchanges were included in our model, and between-colonies exchanges on the same island could also occur. Taking into account on-island displacements should not have an important effect on the persistence index, since the caves CB and CC have very similar epidemiological dynamics.

We assumed the system was closed for EBLV-1 circulation, and in particular, that it was independent of the transmission cycles on the mainland. To date, we have no evidence of exchanges between bat populations on the mainland and those in the Balearic Islands. Serra-Cobo and collaborators have ringed 6369 individuals of *M. schreibersii* on the mainland during the period 1984–2012, and 2123 in the Balearic Islands during the period 1995–2012, and no exchanges between the mainland and the Balearic Islands have been recorded. These observations suggest that if bat exchanges with the continent exist, they are probably scarce. Moreover, the system of caves and bat subpopulations considered does not represent all Balearic Island bat colonies: other bat species (including *E. serotinus*) and other colonies exist, but little is known about them. Obviously, if the subpopulations included in our model were to interact strongly with other bat subpopulations, our results could be affected. Finally, it should be noted that, in the Balearic Islands, no mammals other than bats are known to be infected with EBLV-1.

Several assumptions were made to compensate for unknowns in the natural history of EBLV-1 infections. Most of them were based on *M. myotis* fieldwork [13] because information on the other three species is lacking. First, because *M. myotis* seroprevalence rates did not differ significantly between adults and juveniles, and males and females [13], we did not stratify any population by age or sex. Second, because EBLV-1 infection does not increase the *M. myotis* mortality rate [13], a constant mortality rate was applied across the epidemiological states. This assumption is also well supported by evidence of survival after EBLV-1 infection in *E. serotinus* [24] and other bat species [11]. Third, we assumed that recovered individuals lose immunity and became susceptible again. Repeated sampling via mark-recapture showed that seropositive individuals became seronegative at some later point [19]. Finally, no attempt was made to fit the model to data, because of the scarcity of data on *M. schreibersii* and *M. capaccinii* subpopulations, but this is an important perspective of this work. Nevertheless, our model is able to generate local epidemiological dynamics similar to reported data and our results are strongly supported by global sensitivity-analysis outcomes.

To our knowledge, this is the first attempt to model *Lyssavirus*-transmission dynamics within a metapopulation system including several bat species. This approach provides a general modelling framework that can easily be adapted to different settings with other bat species having a metapopulation structure, and to other zoonotic viruses of public health concern circulating in bat populations. Moreover, we introduced a new approach to analyse stochastic endemic fade-out that is classically quantified through the computation of the expected time to extinction. Our method is more time-efficient, as it avoids running simulations until virus extinction. Indeed, it consists of fitting a simple exponential function to the decreasing trend of the proportion of simulations showing virus persistence over time. Finally, we used the Sobol method (a global sensitivity analyses technique) to identify influential model parameters and rank them in order of importance. This method has rarely been used in infectious diseases modelling, probably due to its high computational cost [25]. It has important advantages however compared to the widely used local One-At-a-Time methods: it is independent from the model structure, it captures the effects of individual parameters and also the effects of their interactions, and it provides quantitative information on the contribution of each parameter to the variance of the output [25].

In conclusion, our model findings improve the understanding of *Lyssavirus* persistence in bat colonies, identifying factors playing key roles in a particular system of multispecies colonies in the Balearic Islands. Our findings suggest that EBLV-1 is sustained in that region because of intra- and interspecies infectious contacts during the breeding and winter periods, and support the hypothesis that *M. schreibersii* serves as a regional reservoir. Moreover, *M. schreibersii*'s immune- and incubation-period durations were among the most critical factors for EBLV-1 persistence.

## Supporting Information

Figure S1 Bat-species distributions.
(PDF)

Supporting Information S1 Impact of a potentially long *Rhinolophus ferrumequinum* infectious period.
(PDF)

Supporting Information S2 Sobol method: sensitivity indexes.
(PDF)
