# Supplementary material for: Insights into Persistence Mechanisms of a Zoonotic Virus in Bat Colonies Using a Multispecies Metapopulation Model
Source: PLoS One. 2014 Apr 22;9(4):e95610. doi: 10.1371/journal.pone.0095610 (PMC3995755; doi:10.1371/journal.pone.0095610)

Figure S1 Bat-species distributions. Pale beige indicates never observed, while red indicates observed.

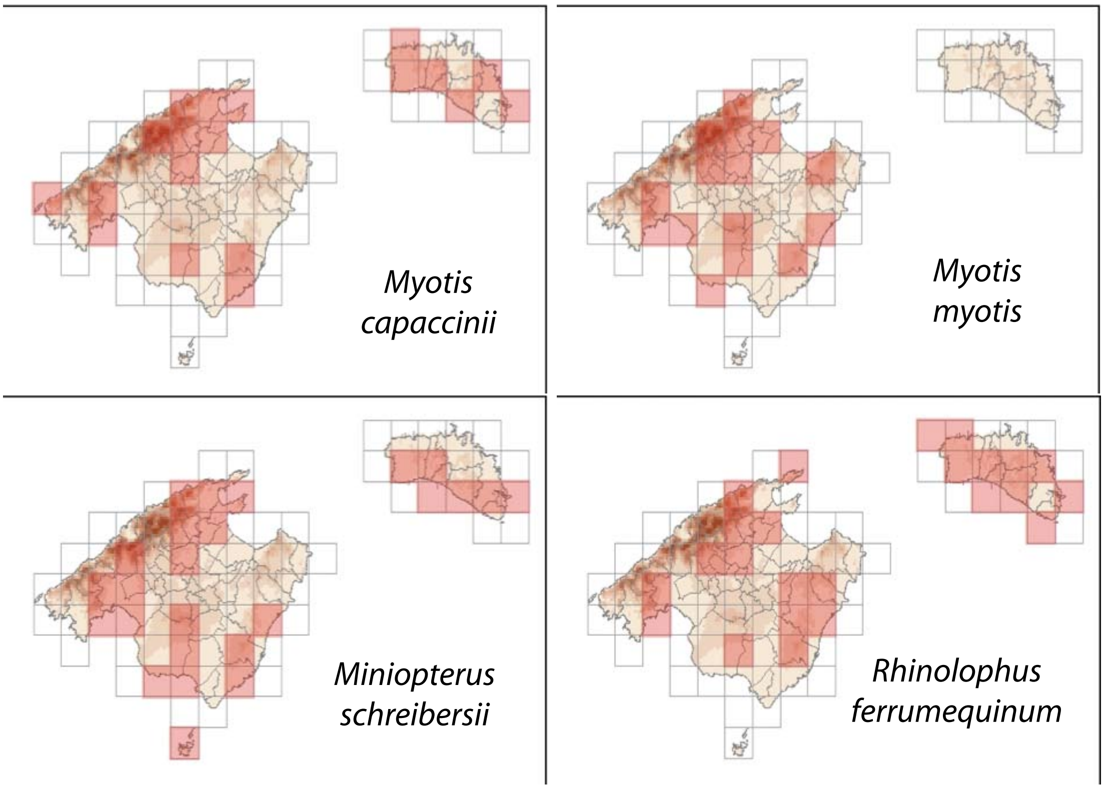

Supplement: Figure S1 — Bat-species distributions. (PDF) [file pone.0095610.s001.pdf]
