# Supplementary material for: Insights into Persistence Mechanisms of a Zoonotic Virus in Bat Colonies Using a Multispecies Metapopulation Model
Source: PLoS One. 2014 Apr 22;9(4):e95610. doi: 10.1371/journal.pone.0095610 (PMC3995755; doi:10.1371/journal.pone.0095610)

Impact of a potentially long *Rhinolophus ferrumequinum* infectious period

To explore how a potentially long *R. ferrumequinum* infectious period could specifically impact our analysis of the roles of ecological factors and each bat species on EBLV-1 persistence on each island, we ran the same analysis now assuming a 50- or 100-day long *R. ferrumequinum* infectious period, instead of 10 days (see Figures S2 and S3).

Because *R. ferrumequinum* presence is greater on Menorca than on Mallorca, virus persistence differed between the islands for these new parameter values. On Menorca, the influence of interisland exchanges was quasi-null, with a clear threshold effect for the influence of contacts during the low-transmission period: $r \leq 0.2$ yielded no persistence, whereas $r \geq 0.6$ yielded maximum persistence. On Mallorca, the effect of contacts during the low-transmission period was also important, but above all, a threshold effect for persistence was seen when the number of individuals traveling between islands exceeded 1: thereafter, the number of individuals did not apparently further influence EBLV-1 persistence. Consequently, removing *R. ferrumequinum* infectious contacts slightly diminished virus persistence on both islands, reaching levels similar to those obtained for a short *R. ferrumequinum* infectious period. When M. schreibersii infectious contacts were removed, EBLV-1 persisted on Menorca because of *R. ferrumequinum* abundance, while on Mallorca, EBLV-1 persistence required seasonal exchanges with Menorca (winter contacts alone did not sustain virus persistence). These observations suggest that, should *R. ferrumequinum* have a longer-than-expected infectious period, M. schreibersii would no longer be the only essential species for EBLV-1 persistence in the system, and moreover, interisland exchanges could be an important factor for EBLV-1 persistence on Mallorca.

The average enzootic period duration was reassessed, now setting R_0 at 3 and r at 0.6, with a *R. ferrumequinum* 100-day infectious period. As before, the M. schreibersii subpopulations had the longest enzootic periods in CB and CC. But, as expected, *R. ferrumequinum* had the longest enzootic period in CA, lasting on average the entire simulation time.

Figure S2 Effect of ecological factors on EBLV-1 persistence when *R. ferrumequinum* has a long infectious period. The persistence index is represented as a function of the seasonal interisland exchanges (Y-axes) and reduction of contacts during low-transmission periods (X-axes), for three different R_0 values (2, 4 and 6). Persistence index shades of gray from white to black correspond, respectively, to low and high probabilities of virus persistence.

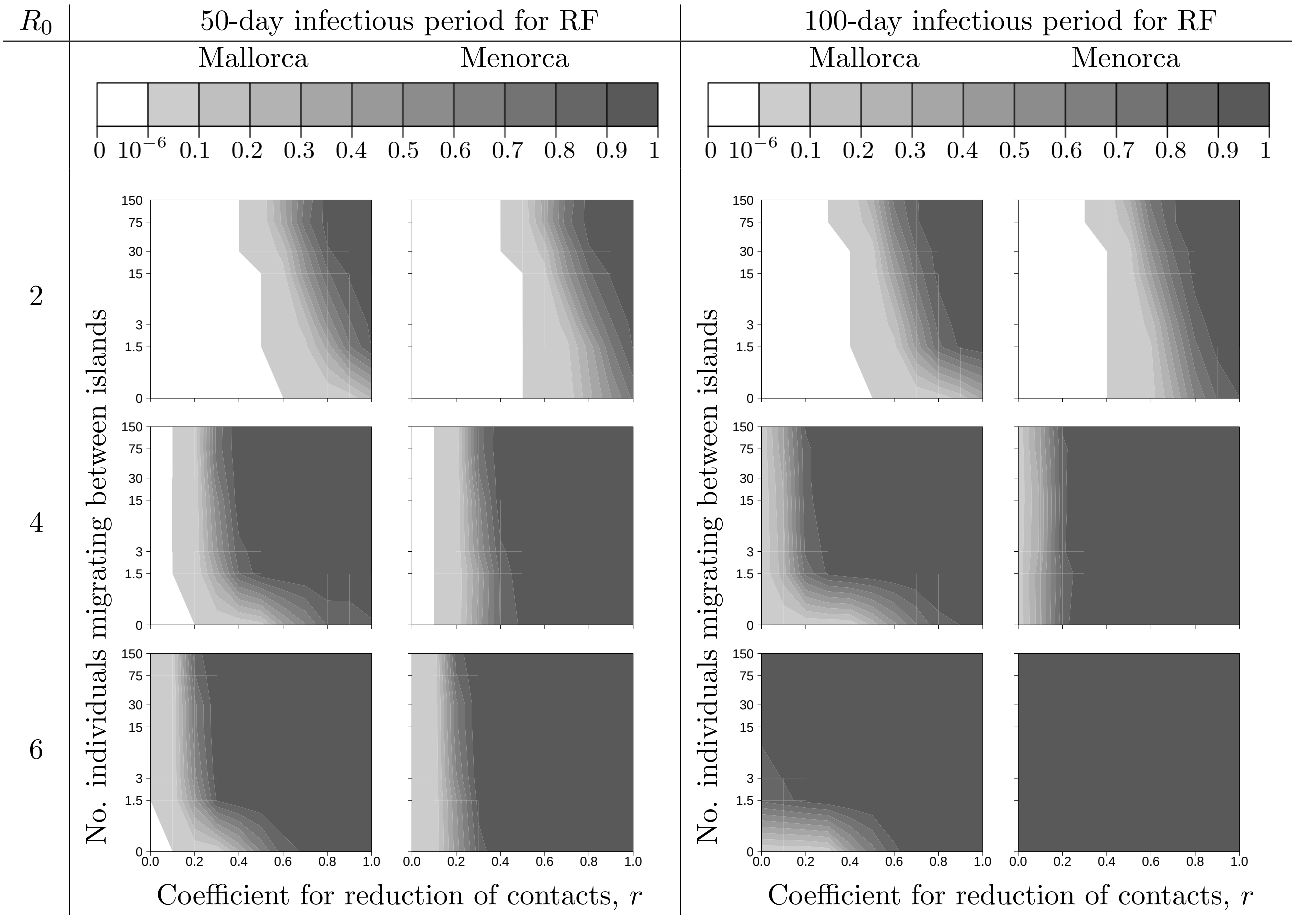

Figure S3 Effect of the different bat species on EBLV-1 persistence when *R. ferrumequinum* has a long infectious period. The persistence index is represented as in Fig. S3. Abbreviations are as given in the legend to Table S3.

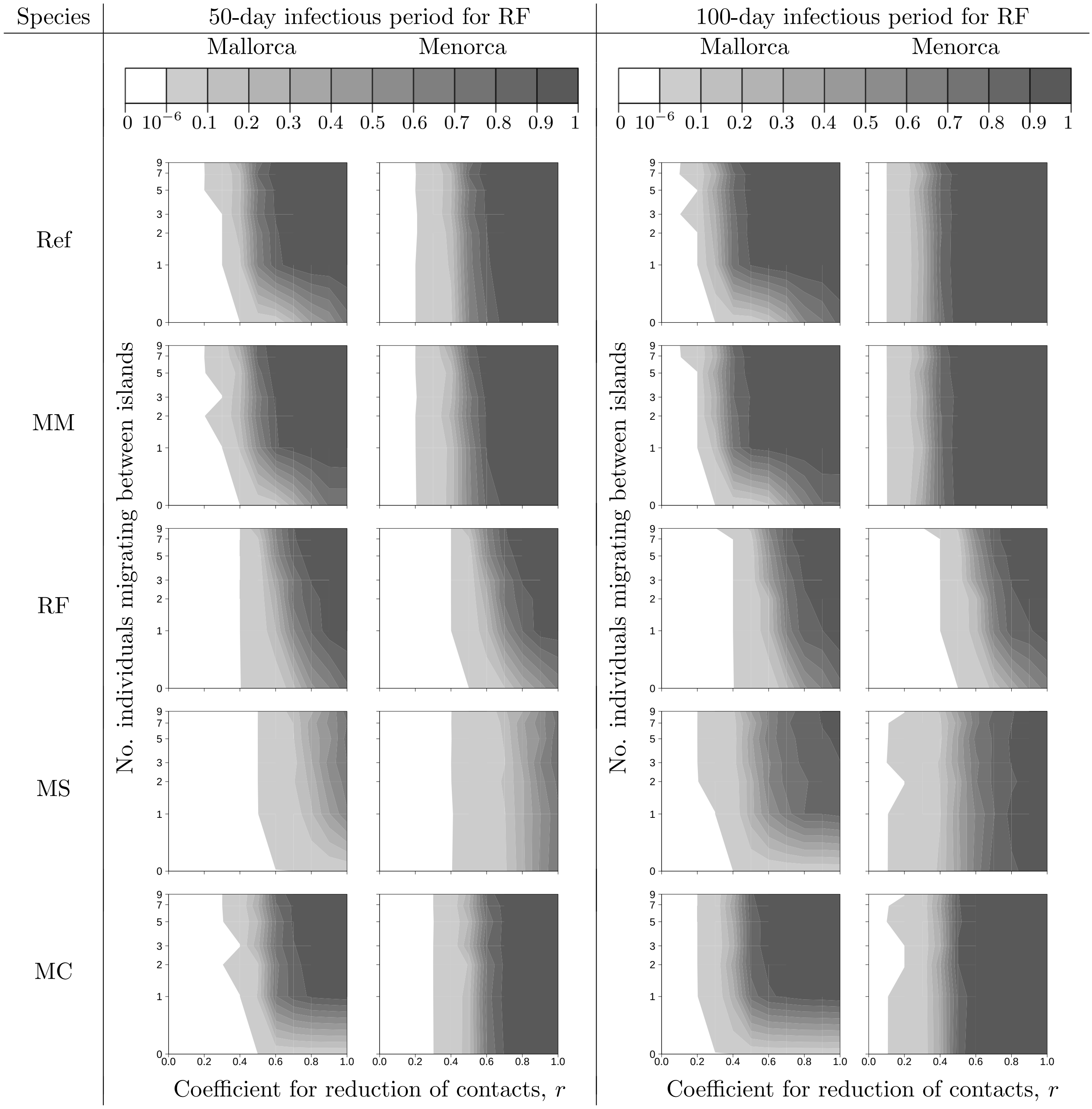

Supplement: Supporting Information S1 — Impact of a potentially long Rhinolophus ferrumequinum infectious period. (PDF) [file pone.0095610.s002.pdf]
