# Supplementary material for: Insights into Persistence Mechanisms of a Zoonotic Virus in Bat Colonies Using a Multispecies Metapopulation Model
Source: PLoS One. 2014 Apr 22;9(4):e95610. doi: 10.1371/journal.pone.0095610 (PMC3995755; doi:10.1371/journal.pone.0095610)
Supplement: Supporting Information S2 — Sobol method: sensitivity indexes. (PDF) [file pone.0095610.s003.pdf]

Sobol method: sensitivity indexes

Consider the following model:

$$Y = f(X_1, .., X_p) \tag{1}$$

where the output Y is a scalar and the input factors $X_1, .., X_p$ are supposed to be independent random variables described by known probability distributions. These distributions reflect the uncertain knowledge on the system. The main idea of this method is to decompose the output variance into the contributions associated with each input factor.

In order to quantify the importance of an input factor X_i on the variance of Y, imagine that we can fix it at its "true" value, x_i^*. How much would this assumption change the variance of Y? This is the conditional variance

$$V_{\mathbf{X_{-i}}}(Y|X_i = x_i^*)$$

where the variance is taken over the $(p-1)$-dimensional parameter space $\mathbf{X_{-i}}$, consisting in all factors but X_i. Because the true value of X_i is unknown, we average over all possible values of X_i:

$$E_{X_i}(V_{\mathbf{X_{-i}}}(Y|X_i))$$

The smaller this quantity, the more important the contribution of X_i to the variance of Y. Indeed, using the law of total variance, we can write:

$$V(Y) = V_{X_i}(E_{\mathbf{X_{-i}}}(Y|X_i)) + E_{X_i}(V_{\mathbf{X_{-i}}}(Y|X_i))$$

and normalizing,

$$1 = \underbrace{\frac{V_{X_i}(E_{\mathbf{X_{-i}}}(Y|X_i))}{V(Y)}}_{S_i} + \frac{E_{X_i}(V_{\mathbf{X_{-i}}}(Y|X_i))}{V(Y)} \tag{2}$$

The first-order sensitivity index for factor X_i is given by the first term in (2):

$$S_i = \frac{V_{X_i}(E_{\mathbf{X_{-i}}}(Y|X_i))}{V(Y)} \tag{3}$$

From (2) we get that the first-order sensitivity index verifies $S_i \leq 1$.

As proved in [1], if the function in (1) is integrable over $[0,1]^p$ then it can be decomposed into terms of increasing dimensionality as follows:

$$f(X_1, .., X_p) = f_0 + \sum_{i=1}^{p} f_i(X_i) + \sum_{1 \leq i < j \leq p} f_{ij}(X_i, X_j) + \cdots + f_{1,\ldots,p}(X_1, \ldots, X_p) \tag{4}$$

Moreover, if the input factors are mutually independent then there exists a unique decomposition of (4) such that all the summands are mutually orthogonal. Using this result, it can be shown that the variance of the output, $V(Y)$, can also be decomposed into:

$$V(Y) = \sum_{i=1}^{p} V_i + \sum_{1 \leq i < j \leq p} V_{ij} + \cdots + V_{1,\ldots,p} \tag{5}$$

where $V_i, V_{ij}, \dots, V_{1,2,\dots,p}$ denote the variance of $f_i, f_{ij}, \dots, f_{1,\dots,p}$ respectively:

$$\begin{aligned}
V_i &= V(E(Y|X_i)) \\
V_{ij} &= V(E(Y|X_i, X_j)) - V_i - V_j \\
V_{ijk} &= V(E(Y|X_i, X_j, X_k)) - V_{ij} - V_{ik} - V_{jk} - V_i - V_j - V_k \\
&\vdots \\
V_{1,\dots,p} &= V(Y) - \sum_{i=1}^{p} V_i - \sum_{1 \leq i < j \leq p} V_{ij} - \dots - \sum_{1 \leq i_1 < \dots < i_{p-1} \leq p} V_{i_1,\dots,i_{p-1}}
\end{aligned}$$

where, for simplicity, the indices for the variance and the mean were omitted.

From this decomposition, sensitivity indexes can be naturally deduced. Note that the first-order indexes defined in (3) can be deduced from the first p terms of the decomposition (5):

$$S_i = \frac{V_i}{V(Y)} = \frac{V(E(Y|X_i))}{V(Y)}$$

The other terms of the decomposition (5) can similarly be interpreted in terms of higher order sensitivity indexes. The second-order sensitivity index, S_{ij}, expresses the amount of variance of Y explained by the interaction of the factors X_i and X_j (i.e. sensitivity to X_i and X_j not expressed in V_i nor V_j)

$$S_{ij} = \frac{V_{ij}}{V(Y)}$$

The third-order sensitivity index, S_{ijk}, expresses the amount of variance of Y explained by X_i, X_j and X_k and not taken into account in the first- and second-order sensitivity indexes

$$S_{ijk} = \frac{V_{ijk}}{V(Y)}$$

and so on until order p. Therefore, for p input factors, we have defined $2^p - 1$ sensitivity indexes. With these definitions of the indexes, we can get the relation:

$$1 = \sum_{i=1}^{p} S_i + \sum_{1 \leq i < j \leq p} S_{ij} + \dots + S_{1,\dots,p}$$

Homma and Saltelli [2] introduced an additional index, the total-order sensitivity index, ST_i, that accounts for all the contributions to the output variation due to factor X_i (i.e. first-order index plus all its interactions):

$$ST_i = \sum_{k\#i} S_k$$

where $\#i$ indicates all the indexes associated to the factor X_i. Using the expressions in (5), it can be shown that this total-order index can be expressed as

$$ST_i = 1 - \frac{V_{\mathbf{X_{-i}}}(E_{X_i}(Y|\mathbf{X_{-i}}))}{V(Y)}$$

and using again the law of total variance and normalizing we get

$$1 = \frac{V_{\mathbf{X_{-i}}}(E_{X_i}(Y|\mathbf{X_{-i}}))}{V(Y)} + \underbrace{\frac{E_{\mathbf{X_{-i}}}(V_{X_i}(Y|\mathbf{X_{-i}}))}{V(Y)}}_{ST_i} \tag{6}$$

Thus the total-order index is given by the second term in (6):

$$ST_i = \frac{E_{\mathbf{X_{-i}}}(V_{X_i}(Y|\mathbf{X_{-i}}))}{V(Y)}$$

Note that the following property can easily be deduced: $0 \leq S_i \leq ST_i \leq 1$.

References

[1] I M Sobol'. Sensitivity estimates for nonlinear mathematical models. *Mathematical Modelling and Computational Experiments*, 1:407–414, 1993.

[2] T Homma and A Saltelli. Importance measures in global sensitivity analysis of nonlinear models. *Reliability Engineering & System Safety*, 52(1):1–17, 1996.
